# Household chaos and screen media use among preschool-aged children: a cross-sectional study

**DOI:** 10.1186/s12889-018-6113-2

**Published:** 2018-10-29

**Authors:** Jennifer A. Emond, Lucy K. Tantum, Diane Gilbert-Diamond, Sunny Jung Kim, Reina K. Lansigan, Sara Benjamin Neelon

**Affiliations:** 1 Department of Biomedical Data Science, Geisel School of Medicine, Dartmouth College, Hinman Box 7920, Williamson Building, 3rd Floor, 1 Medical Center Drive, Lebanon, NH 03756 USA; 2 Department of Epidemiology, Geisel School of Medicine, Dartmouth College, Rubin Building, 8th Floor, 1 Medical Center Drive, Lebanon, NH 03756 USA; 3 Norris Cotton Cancer Center, Rubin Building, 8th Floor, 1 Medical Center Drive, Lebanon, NH 03756 USA; 4 Dartmouth College, 6016 McNutt Hall, Hanover, NH 03755 USA; 5 Department of Health, Behavior and Society, Johns Hopkins Bloomberg School of Public Health, 615 N. Wolfe Street, Baltimore, MD 21205 USA

**Keywords:** Preschoolers, Childhood obesity, Screen media use, Household chaos

## Abstract

**Background:**

Excess screen media use is a robust predictor of childhood obesity. Understanding how household factors may affect children’s screen use is needed to tailor effective intervention efforts. The preschool years are a critical time for obesity prevention, and while it is likely that greater household disorder influences preschool-aged children’s screen use, data on that relationship are absent. In this study, our goal was to quantify the relationships between household chaos and screen use in preschool-aged children.

**Methods:**

A cross-sectional, online survey was administered to 385 parents of 2–5 year-olds recruited in 2017. Household chaos was measured with the Confusion, Hubbub and Order Scale (i.e., the chaos scale), a validated, parent-reported scale. The scale consists of 15 items, each scored on a 4-point Likert scale. Final scores were the sum across the 15 items and modeled as quartiles for analyses. Parents reported their children’s screen use for nine electronic media activities. Adjusted linear and Poisson regression were used to model associations between household chaos and children’s total weekly screen use, screen use within one hour of bedtime and screen use in the bedroom.

**Results:**

Children averaged 31.0 (SD = 23.8) hours per week with screens, 49.6% used screens within one hour of bedtime and 41.0% used screens in their bedrooms. In adjusted regression models, greater household chaos was positively associated with weekly screen use (*P* = 0.03) and use of screens within one hour of bedtime (*P* < 0.01) in a dose-dependent manner. Children in the fourth versus the first quartile of household chaos were more likely to use screens in their bedroom (*P* = 0.03).

**Conclusions:**

Greater household chaos was associated with increased total screen use as well as screen use behaviors that are related to disrupted nighttime sleep. Findings suggest that household chaos may be an obesity risk factor during the preschool years because of such effects on screen use, and highlight the need to consider household chaos when implementing home-based obesity prevention programs for young children.

**Electronic supplementary material:**

The online version of this article (10.1186/s12889-018-6113-2) contains supplementary material, which is available to authorized users.

## Background

Childhood obesity remains a prevalent public health epidemic. Approximately 23% of US preschool-aged children have overweight or obesity [1]. Excess weight in childhood increases the risk for many health problems [2], including non-alcoholic fatty liver disease [3] and type 2 diabetes [4]. Importantly, children who are overweight by the age of 5 are 4–5 times more likely to be obese as adolescents compared to their normal weight peers [5, 6]. Promoting weight loss in children who are already overweight is difficult [7], and excess weight in childhood often tracks to obesity as an adult [8–10]. Given that adult obesity is a considerable risk factor for several comorbidities [11] including type 2 diabetes, heart disease, and several types of cancers [12], preventing excess weight gain during early childhood is an important way to help children achieve optimal health throughout their lifetime.

Excessive screen media use is one of the most robust predictors of childhood obesity. For example, in a meta-analysis of 14 cross-sectional studies [13], each additional hour of TV viewing per day increased the likelihood of obesity by 13% among children. Screen use can affect obesity risk in several ways. Screen use may increase sedentary time and displace physical activity. Screen use may also expose children to advertisements for energy dense foods, which can cue immediate eating [14], and screen use itself may serve as a cue to eat once children begin to pair screen use with eating snacks or meals [15]. Repeated advertisement exposure may also have a longer-term effect on shaping dietary preferences towards calorically dense foods [16]. Furthermore, increased screen use during the preschool years is concerning because excess screen use may negatively affect language development, gross motor skill development and socialization [17]. Strikingly, while the American Academy of Pediatrics recommends that preschool-aged children engage in no more than one hour of high-quality media per day [18], preschool-aged children in the US average 17.5 h per week engaged in screen media [19], nearly 2.5 times the recommended limit.

Screen use may also increase children’s obesity risk because of the negative impacts on nighttime sleep [20–22]. Short sleep (i.e., sleeping less than the recommended minimum 11 h per 24 h for those aged 2 years or 10 h per 24 h for those aged 3–5 years [23]) and disrupted or poor quality sleep are strong predictors of unhealthy weight gain in children [24]. Both screen use before bedtime [25] and screen use in the bedroom [26, 27] relate to disrupted sleep among preschool-aged children, with the mechanisms underlying those relationships including exposure to fearful or scary content [25], delayed bedtimes [28], and exposure to blue-light [29]. Early childhood may even be a unique window during which short sleep has lasting effects on increased fat mass disposition [30] and obesity risk [31] later in childhood. Thus, understanding what household factors may impede the establishment and enforcement of appropriate screen use behaviors during the preschool years may ultimately help parents reduce children’s lifetime risk of obesity.

Household chaos is marked by increased levels of confusion, disorganization and hurriedness [32]. Greater household chaos has been associated with externalizing behaviors in children [33, 34], including attentional and learning problems [35, 36]. Household chaos is also related to poor glycemic control among adolescents with type 1 diabetes [37]. Studies among families with preschool-age children across diverse populations [32, 38] have documented that household chaos is distinct from sociodemographic characteristics. For example, Matheny et al. [32] and Dumas et al. [38] have documented that household chaos is a unique construct that reflects factors such as household traffic, crowding, and lack of order in the home, and is negatively related to parental attention and stress, parenting style and parent-child interactions. While there is considerable research on the construct of household chaos within child development [33–36], less attention has been given to understanding if household chaos impacts children’s obesity risk [39, 40]. Thus, the goal of this study was to assess the associations between household chaos and obesogenic screen use in children aged 2–5 years old, independent of demographic and socioeconomic characteristics. Findings provide novel data on one way in which household chaos may ultimately impact obesity risk in young children.

## Methods

### Study design

In this cross-sectional study, parents of children aged 2–5 years old were recruited via social media during a 5-week period from June to August 2017. Conducting health behavior research via social media is cost-effective, and samples recruited via social media are often more generalizable to the target population as compared to study samples recruited locally [41]. Additionally, the majority of parents in the US use social media (75% in 2014) [42]. For recruitment, advertisements were purchased on Facebook, Twitter and Instagram and were targeted to parents of preschoolers. Parents who clicked the recruitment advertisements were directed to a secure website where they completed a brief screening questionnaire; recruitment advertisements did not disclose the study’s intent. Study eligibility included being a parent/guardian of a child aged 2–5 years old, living with that child at least 50% of the time, and being knowledgeable about that child’s weekly TV use. Parents who completed the survey were invited to enroll in a raffle for one of 20 $50 gift cards to a popular online vendor. All parents provided electronic consent, and the study was approved of by the Committee for the Protection of Human Subjects at Dartmouth College.

### Exposure: Household Chaos

Parents completed the Confusion, Hubbub and Order Scale to assess usual household chaos [32]. This scale includes 15 items to assess confusion, disorganization, and hurriedness in the home (e.g., *We almost always seem to be rushed, You can’t hear yourself think in our home, No matter how hard we try, we always seem to be running late*); no items addressed media use. Each item was scored on a 4-point Likert scale anchored at “very much (4)” to “not at all (1)” like the parent’s home. The final score was a sum of all responses (range 15–60) with a higher score indicating a home that is more chaotic. This chaos scale has high internal consistency (original Cronbach’s alpha = 0.79), is reliable (original test-retest correlation of 0.74), and has been validated against direct observations of parental and household behaviors. Importantly, while scores had low correlations with higher maternal education (Pearson’s *r* = − 0.11) and higher socioeconomic status (Pearson’s *r* = − 0.24) in the original development study, chaos scores were largely distinct from socioeconomic characteristics [32].

### Outcomes: Children’s electronic screen use

Parents completed a series of questions regarding their children’s typical screen use in the past three months. First, parents selected which of nine electronic media activities their children engaged with on any media device; screen use activities were selected based on published reports [43] and the research team’s past experience measuring media use in preschool-age children [14]. Screen activities included 1) watching shows or movies, 2) playing or engaging with apps, 3) viewing videos or clips other than shows or movies online (e.g., YouTube), 4) video calls such as Facetime or Skype, 5) listening to streaming music, 6) browsing or reading electronic books or magazines, 7) playing video games on the Internet, 8) engaging with social media either by self or with another, or 9) browsing websites. For each selected activity, parents reported the usual time per day their children engaged in that activity within the past three months; times were reported separately for a typical weekday and a typical weekend day, and total hours per week were computed for each activity. Because only five and eight children used social media or browsed websites, respectively, those activities were not included in analyses, and total screen use (hours per week) was reported as weekly screen use over the seven remaining activities.

Parents also reported the frequency with which their child used a series of screens (TV, computers, video game consoles, hand-held video game devices, smartphones or touchscreen tablets) within one hour of bedtime (nearly always, often, sometimes, rarely, never); children were considered to usually use screens within one hour of bedtime if parents replied “nearly always” or “often”. Parents also reported if their child used any of those screens in his/her bedroom (yes vs. no).

### Additional covariates

Parents reported their children’s age, gender, race and ethnicity; their own age, education level and relationship to the child; combined annual household income; homeownership status (own, rent or other); and, the number of adults aged 18 years or older (including self), children under the age of 12, and adolescents between the ages of 12 and 17 who live in the home.

### Statistical analyses

Bivariate analyses compared mean household chaos by child, parent and household characteristics using two-sample *t*-tests when comparing means across two groups or one-way ANOVA when comparing means across three or more groups. Linear regression was used to determine the combined associations between child, parent and household characteristics with household chaos, and the adjusted R^2^ value from that model was used to define the proportion of total variance in household chaos explained by the predictors. Children’s total screen use was compared across child, parent and household characteristics using two-sample *t*-tests or ANOVA, as appropriate. Pearson’s correlation coefficients were computed to compare household chaos to total screen use and screen use per each activity. Linear regression was used to fit children’s total screen use on household chaos, adjusted for child age, child race, child ethnicity, child gender, parent age, parent education level, annual household income, homeownership status, the number of children under the age of 12 years in the home, and the number of adults ≥18 years in the home. Those covariates were selected a priori or were measures associated with household chaos or children’s total screen use in bivariate analyses at the *P* < 0.10 level. For the binary outcomes, Poisson regression with robust standard errors [44] was used to estimate the relative risk of screen use within one hour of bedtime and screen use in the bedroom by household chaos, also adjusted for the aforementioned covariates. In all regression models, household chaos was modeled as ranked quartiles, and linear trends over quartile-specific point estimates were assessed; *P* < 0.05 was the threshold for statistical significance for all main effects. Model assumptions were confirmed by summarizing and plotting model residuals. All analyses were completed using the R Language and Environment for Statistical Computing, version 3.3.2.

## Results

### Sample characteristics and household chaos

Of the 1092 parents who completed the screening questionnaire, 519 were eligible and 479 enrolled. Twelve entries were excluded for potentially being from the same household. Of the remaining 467 participants, 385 (82.4%) completed the survey. Median time to complete the survey was 24 min. In the final sample, children’s mean age was 3.3 years (SD = 1.1), approximately half (48.8%) of children were female and most (72.5%) were white, non-Hispanic (Table 1). Household chaos was inversely associated with annual household income (*P* = 0.04), differed by home ownership status (*P* < 0.001), and was positively associated with the number of children in the home under the age of 12 years (*P* < 0.001; Table 1). A linear regression model fitting household chaos on annual household income, home ownership status and number of children in the home under the age of 12 combined explained only 9.4% (adjusted R^2^ = 0.094) of the total variance in household chaos.

**Table 1:** Distribution of sample characteristics and a comparison of mean household chaos by those characteristics

	n (%)	Household chaos	
		Mean (SD)	*P*-value
Overall	385 (100%)	30.1 (8.1)	–
Child characteristics			
Age, years, n (%)			
2	114 (29.6%)	28.9 (7.9)	0.18
3	103 (26.8%)	31.3 (8.5)	
4	97 (25.2%)	30.5 (8.5)	
5	71 (18.4%)	30.0 (7.0)	
Gender, n (%)			
Female	188 (48.8%)	29.6 (8.1)	0.19
Male	197 (51.2%)	30.7 (8.0)	
Ethnicity, n (%)			
Hispanic or Latino	33 (8.6%)	28.9 (6.8)	0.42
Not Hispanic or Latino	334 (86.8%)	28.4 (8.8)	
Did not want to answer	18 (4.7%)	30.3 (8.2)	
Race, n (%)			
White	300 (77.9%)	30.1 (7.9)	0.98
Black or African American	30 (7.8%)	30.1 (8.3)	
Asian	12 (3.1%)	31.1 (6.2)	
Other^a^	43 (11.2%)	29.9 (9.6)	
Parent characteristics			
Age, years, n (%)			
18–29	139 (36.1%)	29.1 (8.2)	0.08
30–39	229 (59.5%)	30.9 (7.9)	
40–49	17 (4.4%)	28.7 (8.9)	
Education level, n (%)			
High school or less	138 (35.8%)	31.4 (8.8)	0.09
Associate’s degree	53 (13.8%)	30.1 (8.5)	
Bachelor’s degree	117 (30.4%)	29.7 (7.2)	
Graduate or professional school	77 (20.0%)	28.6 (7.6)	
Relationship to child			
Mother	365 (94.8%)	30.2 (8.1)	0.74
Father	16 (4.2%)	29.3 (9.3)	
Other	4 (1.0%)	27.5 (6.1)	
Household characteristics			
Annual household income, n (%)			
Less than $25,000	51 (13.2%)	31.2 (9.4)	0.04
$25,000–$64,999	158 (41.0%)	31.2 (8.7)	
$65,000–$144,999	134 (34.8%)	29.1 (7.0)	
$145,000 or more	17 (4.4%)	26.1 (5.5)	
Did not want to answer	25 (6.5%)	29.4 (7.4)	
Home ownership status, n (%)			
Own	214 (55.6%)	29.1 (7.3)	< 0.001
Rent	146 (37.9%)	30.8 (8.5)	
Other	25 (6.5%)	35.5 (10.0)	
Adults (≥18 years) in the home, n (%)			
1	22 (5.7%)	27.7 (7.7)	0.12
2	263 (68.3%)	29.9 (8.2)	
3 or more	100 (26.0%)	31.3 (7.9)	
Children under the age of 12 years in the home, n (%)			
1	81 (21.0%)	27.6 (8.2)	< 0.001
2	169 (43.9%)	29.8 (7.2)	
3 or more	130 (33.8%)	32.4 (8.5)	
Adolescents aged 12–17 years in the home, n (%)			
0	329 (85.5%)	30.0 (7.9)	0.43
1	38 (9.9%)	31.3 (9.8)	
2 or more	10 (2.6%)	27.7 (8.4)	

Among 385 parents with preschool-aged children recruited via social media. *P*-values from ANOVA assessments except for gender where a two-sample *t*-test was used

^a^Other race includes American Indian, Alaskan Native, Pacific Islander, and other

### Children’s screen use and household chaos: Unadjusted associations

Children spent an average of 31.0 (SD = 23.8) hours per week engaged with screens. In unadjusted bivariate analyses, no child characteristics were related to children’s total screen use (data not shown). However, parent age and education level, annual household income, home ownership status, the number of adults in the home and the number of children under the age of 12 years in the home were each associated with children’s total screen use (Additional file 1: Table S1). Household chaos was positively associated with children’s total screen use (*P* < 0.001), watching shows or movies (*P* < 0.001) and viewing online videos (*P* < 0.05; Table 2).

**Table 2:** Preschool-aged children’s screen use and reading by level of household chaos

	Any screen use n (%)	Weekly screen use Mean (SD)	Correlation with household chaos^a^
Total screen use	383 (99.5%)	31.0 (23.8)	0.17†
By type of electronic media activity			
Watching shows or movies	322 (83.6%)	14.7 (12.1)	0.19†
Using apps	253 (65.7%)	4.8 (7.3)	0.08
Viewing online videos (e.g., YouTube)	236 (61.3%)	5.5 (8.7)	0.13*
Video calls (e.g., Facetime, Skype)	137 (35.6%)	1.2 (3.8)	−0.01
Listening to streaming music	100 (26.0%)	2.2 (5.9)	−0.01
Browsing e-books	61 (15.8%)	0.9 (3.6)	−0.07
Playing Internet video games	38 (9.9%)	0.8 (3.4)	0.10

*P < 0.05; †*P* < 0.001

Among 385 parents with preschool-aged children recruited via social media

^a^Pearson’s correlation coefficient

Overall, 194 (50.4%) children engaged with screens within one hour of going to bed and 158 (41.0%) used screens in their bedrooms. Mean household chaos was significantly greater among children who used screens within one hour of going to bed (31.7 [SD = 8.2] vs. 28.5 [SD = 7.7]; *P* < 0.001) and who used screens in the bedroom (31.4 [SD = 8.9] vs. 29.2 [SD = 7.4]; *P* = 0.01) compared to children who did not.

### Children’s screen use and household chaos: Adjusted associations

In the adjusted linear regression model (Table 3), household chaos was linearly associated with children’s total media use (*P* = 0.03). For example, children in the fourth quartile of household chaos averaged 14.4 (95% CI: 7.8, 21.0) more hours per week of screen use than children in the first quartile of household chaos. Predicted means for weekly screen use from that adjusted model were 23.3, 31.0, 32.4 and 37.7 h per week for quartiles 1, 2, 3 and 4, respectively.

**Table 3:** Adjusted associations between household chaos and obesogenic screen use in preschool-aged children

			Outcome^a^					
	Median	n	Weekly screen use (hours)^b^		Screen use within an hour of bedtime^c^		Screen use in the Bedroom^c^	
			beta (95% CI)	*P*-value	RR (95% CI)	*P*-value	RR (95% CI)	*P*-value
Household chaos score, quartiles								
Quartile 1: < 24	21	104	0 (Reference)		1.00 (Reference)		1.00 (Reference)	
Quartile 2: 25–29	27	91	7.7 (1.4, 14.1)	0.02	1.28 (0.92, 1.79)	0.14	1.15 (0.81, 1.62)	0.44
Quartile 3: 30–35	32	96	9.1 (2.7, 15.6)	< 0.01	1.42 (1.04, 1.95)	0.03	0.84 (0.58, 1.22)	0.35
Quartile 4: > 35	40	94	14.4 (7.8, 21.0)	< 0.001	1.69 (1.25, 2.29)	< 0.001	1.43 (1.04, 1.97)	0.03
*P* for linear trend^d^			*P =* 0.03		*P <* 0.01		*P* = 0.42	

Among 385 parents with preschool-aged children recruited via social media

^a^All models also adjusted for child age, race, ethnicity and gender, parent age and education level, annual household income, homeownership status, and the number of adults and children under the age of 12 years in the home

^b^Adjusted beta coefficient from a linear regression model. Adjusted R^2^ = 0.17

^c^Adjusted relative risk (RR) and 95% confidence interval from a Poisson regression model with robust standard error estimates

^d^*P* for linear trend based on simple linear regression fitting point estimates on quartile medians

Greater household chaos was also linearly related to an increased risk of screen use within one hour of bedtime (*P* < 0.01). In comparison, an increased risk of screen use in the bedroom was limited to those in the highest versus the lowest quartile of household chaos (adjusted RR: 1.43; 95% CI: 1.04, 1.97).

## Discussion

The objectives of this study were to assess the associations between household chaos and measures of screen use that are related to an increased obesity risk among preschool-aged children. In the study sample of 385 parents of 2–5 year-olds, household chaos as measured with a validated scale was positively associated with greater total weekly screen use, screen use within an hour of bedtime, and screen use in the bedroom, independent of child, parent and household factors. Study findings document the considerable influence household chaos may have on screen use among preschool-aged children, and highlight the need to understand underlying mechanisms linking household chaos to children’s screen use in order to appropriately tailor obesity prevention interventions for young children.

In the current study, children from homes in the highest quartile of household chaos averaged 14 more hours of screen use per week compared to children in the lowest quartile of household chaos. Household chaos was associated with parental education, annual household income, home ownership status and the number of children in the home, similar to the original development study for the chaos scale [32] in which household chaos was inversely correlated with maternal education and socioeconomic status. However, in the original study and this current study, socioeconomic factors combined explained only a small portion of the total variance in household chaos. Thus, it is likely that household chaos is a unique factor that influences children’s screen use. It is possible that parents in more chaotic homes may be less inclined to monitor and manage children’s screen use because of other demands. Adults in more chaotic households may also be more likely to use media to occupy children; for example, children from more chaotic homes have increased externalizing behaviors including attentional or behavioral problems [33–36], and parents may be more likely to use screens as a pacifying device. Additionally, children in chaotic homes may turn to media for respite from disorder. Qualitative studies are warranted to examine the factors that underlie the relationship between household chaos and greater levels of screen use in preschool-aged children.

Another interesting finding from the current study is that children from households in the highest quartile of household chaos were 69% more likely to use screens within one hour of bedtime and 43% more likely to use screens in the bedroom as compared to children from homes in the lowest quartile of household chaos scores. Findings suggest that increased household chaos may ultimately disrupt sleep during the preschool years directly via screen use, increasing a child’s obesity risk. In a cross-sectional study enrolling 72 low-income Hispanic or African-American families with preschoolers [45], greater family chaos was related to increased bedtime resistance (e.g. difficulty going to bed and falling asleep) and disrupted nighttime sleep among children, although the study did not focus on screen use as a potential mediating variable. Increased household chaos was also related to greater sleep problems among 6–13 year olds [39] and 11–12 year olds [46]. Prospective studies to examine the relationships between household chaos, screen use and sleep during the preschool years are warranted.

The American Academy of Pediatrics recommends no more than one hour of high-quality screen use per day for children 2–5 years old [18]. In the current study, children averaged 2.1 h per day watching TV shows or movies, and an additional 2.3 h per week in other screen use. While excessive screen use is a clear obesity risk factor, increased screen use during the preschool years is concerning for several other reasons [17]. Time spent with screens may displace time when children engage in adult conversation or play with other children, which may affect language development, gross motor skill development and socialization. High levels of screen use is also related to poor self-regulation among preschool-age children [17]. Overall, these associations are concerning given that they relate to reduced school readiness and poor school performance [47]. Study findings also demonstrate that preschool-age children are accessing media content online, highlighting the importance of monitoring online screen use even at a young age. Overall, findings highlight the continued need for home-based programs to shape appropriate screen use behaviors during the preschool-years, particularly for children in more chaotic households. Additionally, parents and caregivers from more chaotic households may benefit from tailored support strategies that address the specific barriers they face when enacting and enforcing appropriate screen use behaviors for their young children.

### Study limitations

In this study, enrolled parents were recruited via social media, therefore, findings may not be generalizable to parents who do not use social media. Also, because parents were social media/online users, children may have been exposed to occasions where they see and mimic their parents using new media, and that may have increased children’s likelihood of screen use. For example, children in this study averaged 31 h of screen use per week, greater than the 17.5 h per week average from a 2017 US nationally-representative survey administered by Common Sense Media [19] yet similar to 2009 values reported by the Nielsen Company (US) of 32 h per week [48]. Parents’ estimates of screen use may not have included children’s use when children were under the care of others, and the study was conducted during summer months, a time when children’s screen use may have been greater if routine child care (e.g., preschool) was not offered during that time. Despite these limitations, this study makes a considerable contribution to the literature by documenting a strong association between household chaos and screen use among preschool-aged children, and additional studies to examine which aspects of household chaos most impact children’s media use are warranted. That line of research may identify how interventions could be best tailored to support more chaotic homes in consistently supporting healthy media use behaviors among young children.

## Conclusions

In this study of parents of preschool-aged children recruited nationally via social media, household chaos was positively associated with total screen use, screen use within an hour of bedtime and screen use in the bedroom, independent of child, parent, and household characteristics. Studies to examine if household chaos, via increased screen use, impacts sleep and other health behaviors including physical activity and dietary behaviors during the preschool years are warranted. Additionally, the current study examined screen use quantity, and studies to examine if household chaos relates to screen use quality among preschool-aged children are needed. Household chaos is multidimensional. Because children’s media use in the preschool-years has many negative lasting effects, intervening quickly on inappropriate media use during this time is needed. Given the findings from this study, practitioners should intervene on children’s media use in a way that is sustainable for those in more chaotic homes. However, programs to reduce household chaos overall are critical to improve child health.

## Additional file

Additional file 1: **Table S1.** Weekly screen use among preschool-aged children by parent and household characteristics. This table presents the distribution of children’s weekly media use (hours per week) by sample characteristics. (DOCX 46 kb)

## Abbreviations

ANOVA: Analysis of Variance
TV: Television
US: United States
