# Supplementary material for: Household chaos and screen media use among preschool-aged children: a cross-sectional study
Source: BMC Public Health. 2018 Oct 29;18:1210. doi: 10.1186/s12889-018-6113-2 (PMC6206857; doi:10.1186/s12889-018-6113-2)
Supplement: Supplementary file 1 — Table S1. Weekly screen use among preschool-aged children by parent and household characteristics. This table presents the distribution of children’s weekly media use (hours per week) by sample characteristics. (DOCX 46 kb) [file 12889_2018_6113_MOESM1_ESM.docx]

**Table S1.** Weekly screen use among preschool-aged children by parent and household characteristics.

|  | **n** | **Weekly media use (hours)**  **Mean (SD)** | | ***P*-value** |
| --- | --- | --- | --- | --- |
| Overall | 385 |  | 31.0 (23.8) | -- |
| Parent characteristics |  |  |  |  |
| Age, years |  |  |  |  |
| 18-29 | 139 |  | 35.6 (23.5) | 0.002 |
| 30-39 | 229 |  | 27.5 (23.1) |  |
| 40-49 | 17 |  | 39.4 (27.1) |  |
| Relationship to child |  |  |  |  |
| Mother | 365 |  | 31.0 (23.7) | 0.69 |
| Father | 16 |  | 28.8 (17.5) |  |
| Other | 4 |  | 40.4 (51.5) |  |
| Parent education level |  |  |  |  |
| High school or less | 138 |  | 40.4 (26.8) | <0.001 |
| Associate’s degree | 53 |  | 28.1 (22.1) |  |
| Bachelor’s degree | 117 |  | 25.2 (19.2) |  |
| Graduate or professional school | 77 |  | 24.7 (20.0) |  |
|  |  |  |  |  |
| Household characteristics |  |  |  |  |
| Annual household income |  |  |  |  |
| Less than $25,000 | 51 |  | 42.8 (35.7) | <0.001 |
| $25,000-$64,999 | 158 |  | 33.0 (21.5) |  |
| $65,000-$144,999 | 134 |  | 26.8 (19.9) |  |
| $145,000 or more | 17 |  | 15.1 (14.0) |  |
| Refused to answer | 25 |  | 27.3 (20.1) |  |
| Home ownership status |  |  |  |  |
| Own | 214 |  | 27.7 (20.4) | 0.002 |
| Rent | 146 |  | 36.5 (27.7) |  |
| Other | 25 |  | 27.1 (20.5) |  |
| Adults (≥18 years) in the home |  |  |  |  |
| 1 | 22 |  | 47.4 (42.3) | <0.001 |
| 2 | 263 |  | 28.2 (19.5) |  |
| 3 or more | 100 |  | 34.7 (26.6) |  |
| Children under the age of 12 years in the home |  |  |  |  |
| 1 | 81 |  | 37.3 (29.8) | 0.03 |
| 2 | 169 |  | 29.0 (21.8) |  |
| 3 or more | 130 |  | 29.6 (21.8) |  |
| Adolescents age 12-17 years in the home |  |  |  |  |
| 0 | 329 |  | 30.7 (23.4) | 0.56 |
| 1 | 38 |  | 34.3 (28.8) |  |
| 2 or more | 10 |  | 26.1 (19.6) |  |

Note: Among 385 parents with preschool-aged children recruited via social media. *P*-values from one-way ANOVA.
